# Protective Role of Diatomite Against Freezing Stress in *Hordeum vulgare* L.: Insights into Physiological Mechanisms

**DOI:** 10.3390/biom16060896

**Published:** 2026-06-17

**Authors:** Saltanat Nayekova, Vladimir Kiyan, Zhanar Tulegenova, Timur Savin, Evgeniy Ten, Zerekbay Alikulov

**Affiliations:** 1 Department of Biotechnology and Microbiology, L.N. Gumilyov Eurasian National University, Astana 010000, Kazakhstan; naekova_sk@enu.kz (S.N.); tulegenova_zha@enu.kz (Z.T.); alikulov_za@enu.kz (Z.A.); 2 Laboratory of Biodiversity and Genetic Resources, National Center for Biotechnology, Astana 010000, Kazakhstan; 3 Scientific Center for Biological Research, Astana 010000, Kazakhstan; 4 A.I. Barayev Research and Production Centre for Grain Farming, Shortandy 020000, Kazakhstan; savintimur_83@mail.ru (T.S.); jekon_t87.07@mail.ru (E.T.)

**Keywords:** diatomite, *Hordeum vulgare*, freezing stress, silicon, antioxidant enzymes, proline, seed priming, plant stress tolerance, photosynthetic pigments, abiotic stress

## Abstract

Freezing stress is one of the major abiotic factors limiting plant growth and productivity. This study evaluates the effects of diatomite (DTM) as a natural silicon-rich amendment on growth performance, physiological responses, and cold stress tolerance in barley (*Hordeum vulgare* L.). Seed priming and substrate application of DTM at different concentrations (5–20%) were used to assess morphological, biochemical, and ultrastructural changes under normal and low-temperature conditions. Results showed that DTM significantly enhanced root growth and biomass accumulation, with the most pronounced effect at 10% concentration. Treated plants exhibited improved survival under freezing stress, along with better preservation of leaf cellular structure and photosynthetic pigments. Biochemical analyses revealed reduced proline accumulation and decreased activity of key antioxidant enzymes, indicating alleviation of oxidative stress and improved redox balance. Electron microscopy confirmed the integration of diatomite particles into seed and tissue structures, providing physical reinforcement and thermal protection. Overall, diatomite acts as a multifunctional, environmentally safe soil amendment that enhances plant growth and improves tolerance to cold stress through combined physical and physiological mechanisms.

## 1. Introduction

Abiotic stress, such as freezing temperatures, has devastating effects on plants. Frost damage causes plants to lose turgor, and the leaves turn brown and wither. Ice primarily forms in the intercellular spaces of the crystals, causing irreversible denaturation of the cell protoplast colloids and tissue death. If only a small amount of ice forms, plants may survive thawing [1,2]. At low temperatures, the destruction of photosynthetic pigments leads to electron leakage and a sudden release of reactive oxygen species (ROS) [3].

The application of natural soil amendments represents a sustainable approach in modern agronomy to bolster plant stress tolerance and minimize environmental impacts. Unlike synthetic fertilizers, natural inputs do more than just deliver essential nutrients; they actively enhance the soil’s physical, chemical, and biological structure, fostering beneficial microflora and increasing humus content [3].

Silicon (Si) has emerged as an effective agent for mitigating abiotic stress, including cold and frost damage in plants. Although not classified as an essential metabolic nutrient, silicon application, whether incorporated into the soil or used as a seed-priming treatment, significantly reduces oxidative damage, maintains trace element homeostasis, and regulates hormonal balance in stressed crops. This protective effect is especially critical in Northern Kazakhstan, where early spring planting of wheat and barley regularly encounters harsh continental conditions, including sudden frosts as low as −4 °C. Exposure to such freezing temperatures during early growth stages triggers severe cold stress, often leading to substantial crop failure [4,5].

Modern agronomy increasingly relies on natural fertilizers to bolster plant stress resistance and promote sustainability. These natural inputs do more than just supply essential nutrients; they actively enhance the soil’s physical, chemical, and biological properties, fostering a healthy microbiome and enriching the soil ecosystem [6,7,8,9,10,11,12,13].

The mechanism of action involves the activation of antioxidant system genes upon exposure to Si during various abiotic stresses. Unlike bulk silicon, engineered silicon nanoparticles (1–100 nm) offer unique advantages such as a high specific surface area, distinct charge properties, and enhanced bioavailability. They can modulate gene expression and boost antioxidant enzyme activity [14,15,16,17]. In this study, we focused on diatomaceous earth, a natural material that enhances crop yields and abiotic stress tolerance through its physical (low thermal conductivity) [18,19] and chemical properties [20]. Because it works without altering the plant’s genetic machinery, it preserves the vital metabolic energy required for germination and growth. Furthermore, the silanol groups on the surface of diatomaceous earth interact with water molecules to maintain cellular hydration, support protective osmolytes such as proline, and effectively scavenge reactive oxygen species (ROS), including superoxide anions and hydroxyl radicals.

In our previous studies, we demonstrated that treating seeds with diatomaceous earth can increase germination, stimulate seedling growth under saline conditions, and reduce the development of microbial pathogens around the seed, demonstrating potential improvement in plant stress tolerance [21,22]. A study of the chemical composition also indicated the environmental safety of diatomite for use in agronomy [23,24].

Thus, the relevance of this study lies in the fact that Kazakhstan has a huge reserve of diatomite (approximately 3 billion tons) in Western Kazakhstan, which can be used in agronomy to improve crop yields and combat abiotic and biotic stress, as existing results indicate that silicon and silicon-containing natural fertilizers can mitigate stress effects. This study aimed to investigate the mechanism of diatomite particle penetration into seeds during pre-sowing treatment and analyze its effect on cold stress (frost). The data obtained can be applied to the development of new priming methods capable of increasing crop resilience to adverse climatic conditions.

## 2. Materials and Methods

### 2.1. Diatomite Sampling and Characterization

Diatomite samples were collected from the Mugodzhar Hills (Aktobe region, northwestern Kazakhstan), a low-mountain ridge representing the southern continuation of the Ural mountain system.

**Figure 1:**
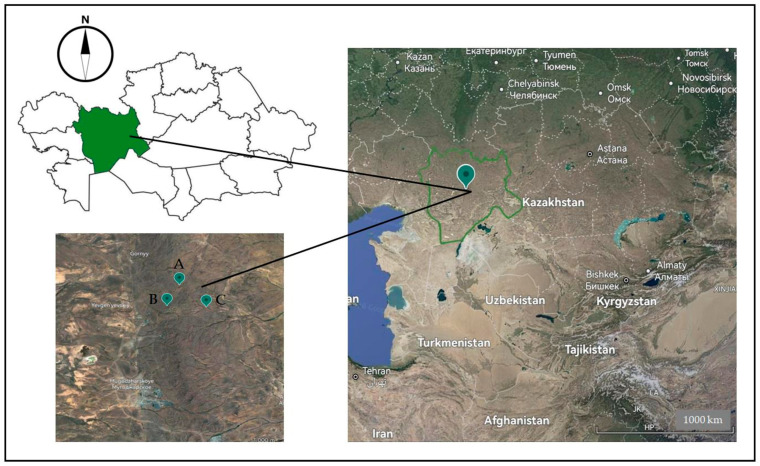
Sampling locations of diatomite in the Mugodzhar Hills (Kazakhstan). Geographical distribution of sampling points: northern Mugodzhar (A), central Mugodzhar (B) and southern Mugodzhar (C).

Sampling was conducted at three geographically distinct points (Figure 1): Point A (48°38′56″ N, 58°32′45″ E)—northern Mugodzhar; Point B (48°38′08″ N, 58°31′29″ E)—central Mugodzhar; Point C (48°37′32″ N, 58°33′55″ E)—southern Mugodzhar. Collected samples were air-dried and used for further structural and experimental analyses.

### 2.2. Diatomite Preparation and Seed Priming Treatment

Natural diatomite (DTM), a sedimentary siliceous material composed primarily of amorphous silicon dioxide and characterized by its high porosity and adsorption capacity, was used as a mineral priming agent. Diatomite was ground to obtain a particle size fraction of 5–60 μm. Aqueous diatomite suspensions were prepared at concentrations of 5%, 10%, and 20% (*w*/*v*) in distilled water. The prepared suspensions were stirred using a magnetic stirrer for 60 min to ensure complete particle distribution. The prepared diatomite suspensions were sterilized by autoclaving at 120 °C and 0.1 MPa for 20 min. Seed priming was performed by immersing sterilized seeds in cooled sterile diatomite suspensions for 24 h under sterile conditions [23,24,25,26].

### 2.3. Plant Material and Growth Conditions

Seeds of *Hordeum vulgare* L. were provided by the certified seed bank of the A. I. Barayev Scientific and Production Center for Grain Farming (Akmola Region, Republic of Kazakhstan). The seeds were surface-sterilized with 2% NaClO and 70% C2H5OH for 2–3 min, followed by three rinses with autoclaved distilled water. Experimental Design and Freezing Stress Protocol: Pots of 250 mL capacity, filled with a 3:1 (*v*/*v*) soil: sand mixture, were sown with 10 seeds each to establish five distinct treatment groups under a completely randomized design. Each treatment was performed in triplicate (*n*). For the initial 7 d, all seedlings were raised uniformly in a climate chamber, maintaining a 16 h light/8 h dark photoperiod at 20 ± 1 °C and were irrigated daily with distilled water. All four groups, specifically a non-pretreated stress control alongside three groups pretreated with 5%, 10%, or 20% DTM suspensions, were transferred to freezing conditions at −3 °C for 6 h after 7 days of planting under normal conditions. The fifth group remained undisturbed at 20 °C to serve as a non-stressed control. The experiment was performed with three biological replicates [23].

### 2.4. Freezing Stress Treatment

The plants were transferred directly from optimal growth conditions to a pre-cooled chamber at −3 °C to induce freezing stress. Temperatures between 0 and 15 °C are generally associated with cold stress, whereas temperatures below 0 °C cause freezing stress. Seedlings were exposed to −3 °C for 6 h. This exposure period was selected to investigate the early physiological and biochemical responses of plants to freezing stress, including the initial phase of reactive oxygen species (ROS) accumulation and membrane permeability changes reported in cereals during the first hours of cold exposure [27]. After the freezing treatment, the plants were allowed to recover for 24 h under optimal growth conditions (20 ± 1 °C, 16 h photoperiod). Seedling survival was evaluated after the recovery period.

### 2.5. Growth and Morphological Analysis

Growth parameters included root length, shoot length, and total biomass. Biomass was determined after drying plant material at 70 °C to constant weight. Seedling survival rate was calculated after cold stress exposure. Morphological observations of leaf tissue were performed using an AxioScope A1 trinocular light microscope (Zeiss, Oberkochen, Germany) under transmitted light conditions.

### 2.6. Analysis of Photosynthetic Pigments

The chlorophyll a, chlorophyll b, and carotenoid contents were determined from the leaf margins of the samples. Plant samples were then incubated in the dark for 24 h in foil-wrapped Eppendorf tubes. After extraction and complete tissue discoloration, chlorophyll a, chlorophyll b, and total carotenoid contents were quantified via spectrophotometry at 665, 649, and 470 nm, respectively. Pigment concentrations (mg/g wet weight) were calculated using the Lichtenhaler formula [28]:

$$Chla=\frac{\left[\left(13.95\cdot D665-6.88\cdot D649\right)\cdot V\right]}{m}$$

$$\;\;Chlb=\frac{\left[\left(24.96\cdot D649-7.32\cdot D665\right)\cdot V\right]}{m}$$

$$Ccar=\frac{\left[\left(1000\cdot D470\cdot \frac{V}{m}-2.05\cdot Chla-114.8\cdot Chlb\right)\right]}{245}$$

V—volume of extract (ml);

m—sample weight (mg);

D—optical density.

### 2.7. Antioxidant Enzyme Determination and ROS Quantification

We harvested fresh root apices and the second fully expanded leaves from seedlings seven days after treatment. The samples were immediately flash-frozen in liquid nitrogen and ground into fine powder. All downstream isolation procedures were performed at 4 °C.

#### 2.7.1. Superoxide Dismutase (SOD) Activity

SOD activity was determined using nitroblue tetrazolium (NBT). It reacts with superoxide anions, which are formed during aerobic action, and reduces the amount of NADH and phenosine metasulfate (PMS). As a result of this reaction, NBT produces hydroxytetrazolium (formazan). In the presence of SOD, the percentage of NBT reduction decreased. After 50% incubation of the NBT reduction reaction, the enzyme activity was expressed in units corresponding to 1 g of the protein. The absorbance of the reaction mixture was measured at 560 nm using a spectrophotometer. The specific activity was expressed in units per milligram of protein (U/mg). The calculation was performed using the following formula [29]:

SOD Activity(U) = [%Inhibition/50]·DF

Dilution factor (DF = 1)

$$\;\;\;Inhibition\left(\%\right)=\frac{\left[Acontrol-Asample\right]}{Acontrol}\times 100\%.$$

A control—optical density of control;

A sample—optical density of sample.

#### 2.7.2. Ascorbate Peroxidase (APX) Activity

APX activity was determined by monitoring the decline in ascorbate absorbance at 290 nm. The enzyme activity was calculated using the extinction coefficient of reduced ascorbate (2.8 mM ^−1^ · cm ^−1^) and expressed in microns of ascorbate/min · g of the raw mass of the roots and shoots. The specific activity was expressed in μmol ascorbate (min^−1^ · g^−1^) [30]:

$$A=\frac{\left[A290\cdot Vtotal\right]}{\left[\varepsilon \cdot d\cdot Vsample\right]}\cdot \frac{1}{m}$$

A290—change in absorbance over 1 min (abs. units/min);

V total—total volume of the reaction mixture (mL);

V sample—volume of extract (sample) added to the mixture (mL);

d—optical path length (of the cuvette), usually 1 cm;

ε—molar extinction coefficient of ascorbate at 290 nm. In most protocols, it is equal to 2.8 mM^−1^ · cm^−1^.

#### 2.7.3. Catalase (CAT) Activity

Catalase (CAT) activity was calculated according to the standard protocol described by Aebi (1984) [31]. The activity was calculated based on the decomposition of H_2_O_2_ monitored at 240 nm using a molar extinction coefficient of 39.4 M^−1^ · cm^−1^. The final specific activity was expressed as μmol H_2_O_2_ · min^−1^ · mg^−1^ protein using the following formula:

$$A=\left[\frac{\Delta A240}{\varepsilon }\right]\cdot \frac{\left[Vtotal\right]}{\left[C\left(protein\right)\cdot Vsample\right]}$$

ΔA 240—change in optical density over 1 min;

V total—total volume of the reaction mixture (mL);

V sample—volume of enzyme extract added to the mixture (mL);

d—optical path length (1 cm);

ε—extinction coefficient of H_2_O_2_ (equal to 39.4).

#### 2.7.4. Proline Content

Plant samples (0.04 g) were homogenized in 3% sulfosalicylic acid. After 72 h, proline was released. The homogenate was centrifuged at 3000 rpm for 20 min [32]. The supernatant was then treated with acetic acid and ninhydrin, boiled for 1 h, and the absorbance was measured at 520 nm using a BioPhotometer basic D30 UV-visible spectrophotometer (Eppendorf, Hamburg, Germany). The proline content was expressed as mg per gram of dry weight (DW):

$$\;\;C=\frac{\left[Cn\cdot V\right]}{\left[115\cdot 5\cdot m\right]}$$

Cn—proline concentration determined from the calibration curve, μg/mL;

V—volume of Toluen (mL);

m—the sample weight of plant material (g).

#### 2.7.5. Protein Content

The total soluble protein content was determined using the Bradford method [33] with bovine serum albumin (BSA) as a standard. One hundred microliters of plant extract was mixed with 5 mL of Bradford reagent (Coomassie Brilliant Blue G-250). After 5–10 min of incubation at room temperature, the absorbance was measured at 595 nm using a spectrophotometer. Protein concentration was expressed as mg per gram of fresh weight (mg/g of FW):

$$C\;=\frac{\left[Asample-A0\right]}{K}$$

C—protein content of the sample (mg/mL);

Asample—optical density of sample at 595 nm;

A_0_—optical density of blank sample;

K—coefficients from the calibration curve equation.

#### 2.7.6. Determination of Superoxide Radical

The visualization of root-localized superoxide anion generation relied on the fluorogenic conversion of dihydroethidium (DHE). The experimental protocol partitioned the root system into three distinct physiological exposure regimes. For temporal tracking, roots were placed in a basal medium containing 0.1 mM KCl and 0.1 mM CaCl_2_ (buffered with 4 mM Tris-HCl, pH 6.0) for 10, 30, 60, or 120 min, followed by a 30 min immersion in 10 μM DHE. Specificity verification involved a 60 min pre-incubation with 5%, 10%, or 20% DTM suspensions to intercept radical activity prior to standard 30 min DHE staining. We placed plants at −3 °C for 6 h, then moved them to a buffer to clear excessive extracellular tracers from all specimens (for quantitative evaluation of DHE fluorescence, *n* = 20 individual observations of ROS from 3 independent biological samples). Epifluorescence documentation was performed using a Nikon Eclipse TS100F with trinocular viewing tube and CFI60 infinity optics (Nikon Corporation, Tokyo, Japan) (excitation: 450–490 nm; emission: 600 nm), and pixel-level intensity was processed using NIS-Elements AR version 5.30 [34].

#### 2.7.7. Hydrogen Peroxide Quantification

Endogenous hydrogen peroxide (H_2_O_2_) mapping was adapted from the colorimetric protocol established by Patterson et al. (1984) [35], as follows: Ice-cold acetone was used as the extraction solvent for the plant matrices. For the peroxytitanium complex formulation, a 3 mL volume of clear extract was treated with 1 mL of 0.1% titanium dioxide (TiO_2_) pre-dissolved in 20% H_2_SO_4_. All insoluble precipitates were separated from the mixture during a 15 min chilled spin at 6000× *g* (4 °C). Finally, the optical density of the remaining stable yellow supernatant was measured at 415 nm using a spectrophotometer. Absolute H_2_O_2_ levels were back-calculated against a 100–1000 µmol standard calibration curve and expressed as µmol g^−1^ fresh weight (FW) using the following equation:

$$H_{2}O_{2}\left(\mu mol\;\cdot \;g^{-1}\right)=\frac{\left[C\;\times \;V\;total\right]}{\left[V\;sample\;\times \;m\right]}$$

C—concentration of H_2_O_2_ in the reaction mixture determined from the calibration curve (µmol);

V total—total volume of the acetone extract (mL);

V sample—volume of the extract used for the reaction (3 mL);

m—fresh weight of the plant sample (g).

#### 2.7.8. SEM-EDS Analysis

A JEOL (Japan) SEM-EDS instrument was used to determine the elemental profiles and map the internal diatomite localization within seed tissues. At 4 °C, microtome-cleaved seed slices underwent 2 h of structural stabilization in 2.5% glutaraldehyde buffered with 0.1 M phosphate (pH 7.2). An ascending 30–100% ethanol gradient was used to dehydrate the rinsed tissues, and a CO_2_ critical-point drying cycle was used to complete the sample matrix preparation. Following the securement of dry sections onto aluminum stubs, a ~10 nm gold sputter layer was deposited. Morphological attributes were documented at an accelerating voltage of 15 kV under a 500–5000× magnification window. Concurrently, 15-kV EDS elemental microanalysis was used to map the spatial distribution and relative mass abundance of diatomite-derived silicon (Si) and oxygen (O) signals [36].

### 2.8. Geometric Modeling of Diatomite Surface Interaction with ROS

The in silico assessment of spatial compatibility and steric alignment between the diatomite substrate and reactive oxygen species (ROS) utilized a cluster-modeling approach. Avogadro software (v. 1.2.0) facilitated the construction of a representative silicon dioxide (SiO_2_) surface matrix terminated with active silanol (Si–OH) groups. Three-dimensional topologies for hydrogen peroxide (H_2_O_2_) and the superoxide anion radical (O_2_•^−^) were built independently. The initial relaxation of all molecular geometries relied on the universal force field (UFF) to establish the baseline valence angles and equilibrium bond lengths. Systematically translating the ROS targets across the silanol coordinates mapped sterically permissible docking conformations. Interatomic distance evaluation using the built-in Avogadro 2.0.0 tools flagged potential hydrogen-bonding networks within a strict d < 2.5 Å threshold. Final spatial inspection and high-resolution rendering were performed using PyMOL (v. 2.5), applying surface modes to display the structural layout of the ROS coordinates over the active diatomite configuration. Stick-and-sphere representations highlight intermolecular contacts to map the steric shielding or trapping mechanics of radicals along the silanol interface [37,38].

### 2.9. Statistical Analysis

We used GraphPad Prism 8.0.1 software, a one-way ANOVA model, with post hoc mean variance isolated using Dunnett’s multiple comparison test. The experimental values represent the mean ± standard error (SE) derived from a minimum of three independent, biological replicates. The threshold for statistical significance was set at *p* ≤ 0.05 [39].

## 3. Results

### 3.1. Microstructural Characterization of Mugodzhar Diatomite

**Figure 2:**
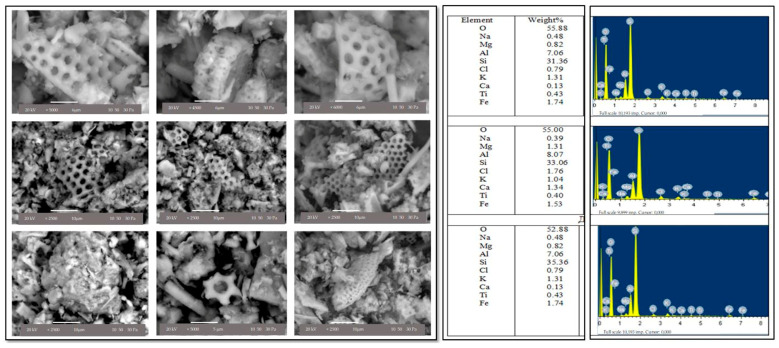
Microstructure of Mugodzhar diatomite samples and their chemical structures (*n* = 3).

All diatomite samples consisted of numerous fragments and whole diatom valves. Microscopic examination of diatomite revealed a unique ultrastructure formed from the skeletons of dead diatoms (Figure 2). Preliminary analysis of the elemental composition of diatomite showed the presence of the following important elements: Mg, Al, Si, K, Ca, Ti, and Fe. In all types of diatomite, the main component of the chemical composition of the rock is silicon dioxide (SiO_2_), with an average content of 70–80%: Si-31–35% and O-55–60%, in smaller quantities—oxides of aluminum and iron (III) (Al-7.06–8%; Fe-1.53–1.74%). The chemical compositions of the diatomite samples were similar.

The size of the diatom frustules ranged from 5 to 60 µm. The structure of diatomite consists of intrafrustrular pores (0.04–0.6 µm), which provide high overall porosity. The sample consisted of well-preserved diatom structures, including fine fragments, which indicated a high degree of structural integrity and suggested a strong adsorption capacity and water-retention potential.

### 3.2. Effects on Morphometric Parameters

**Figure 3:**
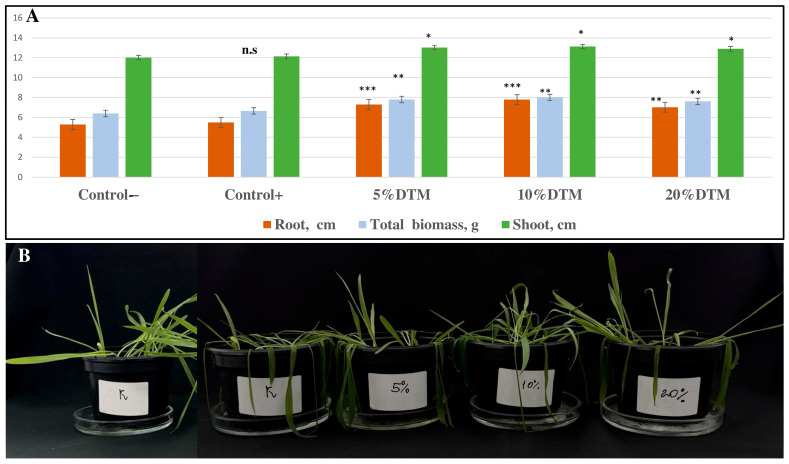
Growth parameters of *Hordeum vulgare* L. on day 7 affected by diatomite priming. Phenotypic responses were evaluated under (**A**) optimal growth conditions and (**B**) freezing stress (-3OC for 6 h). All values are presented as mean ± SE (*n* = 3). *—*p* < 0.05 (differences are statistically significant), **—*p* < 0.01 (high statistical significance), *** *p* < 0.001 (very high statistical significance), n.s—differences are not significant comparatively to (Control−) via one-way ANOVA and Dunnett’s test.

Analysis of the presented data showed that seed priming with DTM had a pronounced stimulating effect on the morphometric parameters of *Hordeum vulgare* L., with the most significant positive effect observed with a 10% DTM concentration (10% DTM) (Figure 3).

According to the histogram (A), DTM treatment at all concentrations (5%, 10%, and 20%) led to a statistically significant (*p* ≤ 0.01) increase in root length, shoot height, and total biomass compared to the negative control, while the positive control (Control+) showed no significant differences from the baseline values (similar variations). Peak growth values were recorded in the 10% DTM variant, where root and shoot lengths reached their maximum values, which correlated with the visual data in panel (B), where plants of this group appeared to be the most developed and robust. When exposed to subzero temperatures (−3 °C) for 6 h, plants pre-treated with DTM retained better leaf turgor and vertical orientation than control samples, which showed clear signs of lodging and thermal tissue damage. The decrease in efficacy with increasing concentrations of 20% DTM indicates a dose-dependent effect of DTM.

An important aspect is the lack of toxicity of DTM, as evidenced by the stable growth and development of plants in all experimental groups without signs of necrosis or chlorosis. Although maximum stimulation of morphometric parameters under normal conditions was characteristic of a 10% concentration, the 20% DTM variant was the most cold-resistant, ensuring the best preservation of vegetative mass at subzero temperatures. Therefore, 10% and 20% DTM concentrations are optimal for pre-sowing treatment, as they act as effective and safe biostimulants that not only accelerate growth but also significantly enhance the adaptive potential of barley to abiotic stress.

### 3.3. Impact of Freezing Stress on Leaf Microstructure

**Figure 4:**
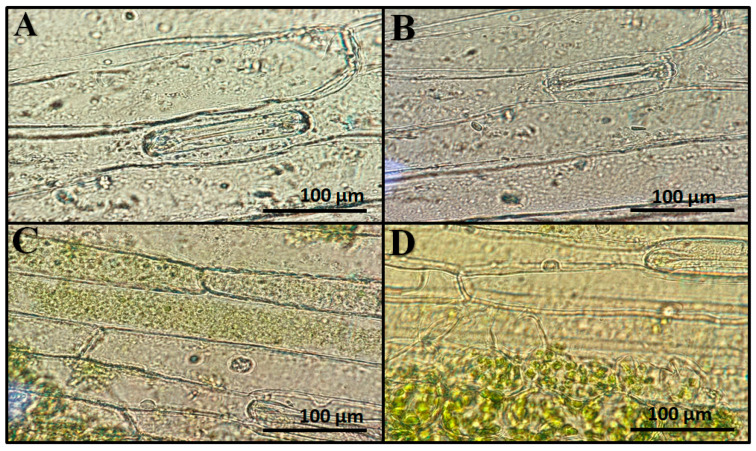
Anatomical features of *Hordeum vulgare* L. leaves under freezing stress (−3 °C, 6 h) following diatomite application: (**A**) Control+; (**B**) 5% DTM; (**C**) 10% DTM; (**D**) 20% DTM.

Microscopic analysis revealed substantial structural differences in leaf tissues between control and DTM-treated plants after cold stress exposure (−3 °C, 7 h) (Figure 4).

In control plants (Figure 4A), tissue disorganization, deformation of mesophyll cells, and disruption of cellular integrity were observed. Treatment with 5% DTM (Figure 4B) partially preserved tissue organization, with more regular cell arrangement and reduced intercellular damage. The most pronounced protective effect was observed at 10% DTM (Figure 4C), where mesophyll cells retained their structural integrity, high density, and regular morphology, suggesting effective protection of the photosynthetic apparatus and water balance. At 20% DTM (Figure 4D), overall tissue structure was maintained; however, localized cell deformation and irregular organization were observed, possibly indicating excessive mineral load.

### 3.4. Effects of Diatomite on Photosynthetic Pigments and Proline Accumulation Under Cold Stress

#### 3.4.1. Photosynthetic Pigments

**Figure 5:**
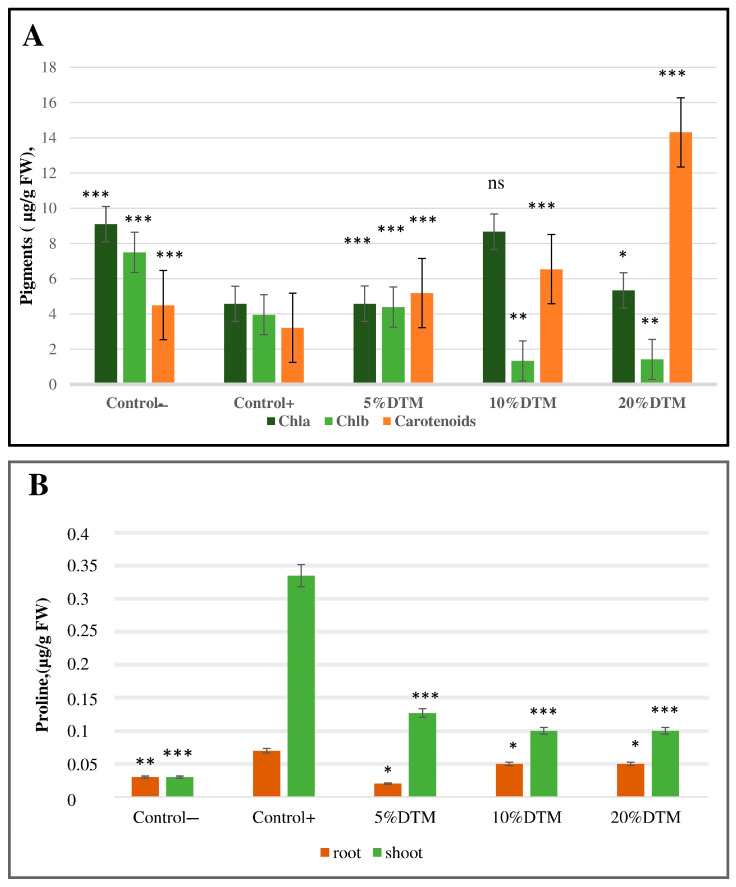
Influence of diatomite application on (**A**) photosynthetic pigment concentrations and (**B**) proline accumulation in *Hordeum vulgare* L. subjected to freezing stress (−3 °C for 6 h). Data points are expressed as mean ± SE (*n* = 3). *—*p* < 0.05 (differences are statistically significant), **—*p* < 0.01 (high statistical significance), *** *p* < 0.001 (very high statistical significance), ns—differences are not significant comparatively to (Control−) via one-way ANOVA and Dunnett’s test.

Although the photosynthetic machinery was markedly compromised by subzero temperatures, DTM application promoted a dose-dependent recovery of pigment concentration (Figure 5A).

Significant pigment degradation was observed in the Control+ group compared to that in the negative control group. However, the application of 5% DTM caused a statistically significant increase (*p* ≤ 0.001) in chlorophyll a (Chl a) content compared to the positive control, indicating the beginning of the stabilization of the light-harvesting complex. Notably, at 10% DTM, Chl a levels reached a plateau, where the difference from the negative control became insignificant, indicating almost complete restoration of this pigment to its physiological norm. In contrast, the dynamics of chlorophyll b (Chl b) showed clear trends in both years. While 5% DTM maintained chlorophyll b levels comparable to the control+ sample, higher concentrations (10% and 20% DTM) resulted in a statistically significant decrease in chlorophyll b content. This decrease in chlorophyll b, an accessory pigment, combined with a simultaneous sharp increase in carotenoid content (peaking at 14.2 μg/g fr. wt. at 20% DTM) suggests the functional restructuring of the photosynthetic antenna. The plant appears to prioritize photoprotection over light absorption, using carotenoids as “screens” to dissipate excess energy and neutralize ROS generated during treatment at −3 °C.

#### 3.4.2. Proline Content

Freezing stress induced a marked accumulation of proline in control plants. In the shoots, the proline content was 0.335 μg/g. Diatomite treatment caused a dose-dependent decrease in proline content in the roots and shoots (Figure 5B). At 10%and 20% concentrations, proline levels in the shoots were reduced to near baseline levels. A similar trend was observed in the roots, where proline content decreased compared with that in the control.

### 3.5. Antioxidant Enzyme Activities and Protein Content

**Figure 6:**
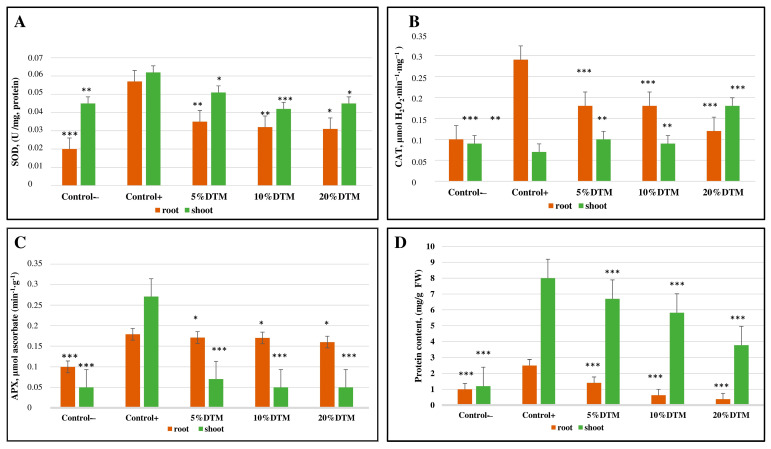
Effects of diatomite on the physiological and biochemical parameters of *Hordeum vulgare* L. under freezing stress: (**A**) SOD activity (U/mg protein); (**B**) CAT activity (µmol H_2_O_2_ min^−1^ mg^−1^ protein); (**C**) APX activity (µmol ascorbate min^−1^ mg^−1^ protein) in roots and shoots; (**D**) total protein content (mg/g). Data are presented as mean ± SD (*n* = 3). *—*p* < 0.05 (differences are statistically significant), **—*p* < 0.01 (high statistical significance), *** *p* < 0.001 (very high statistical significance).

The effects of DTM on antioxidant system parameters and protein content in roots and shoots of *Hordeum vulgare* under cold stress are presented in Figure 6.

#### 3.5.1. Superoxide Dismutase (SOD) Activity

Exposure to low temperatures triggered a distinct surge in SOD activity within the Control+ group, reflecting the intensive accumulation of superoxide radicals under hypotermic stress (Figure 6A). In contrast, DTM application led to a dose-dependent reduction in enzyme activity, bringing it closer to the level of the negative control (Control−). This trend supports the “protective shield” hypothesis, which suggests that DTM likely mitigates stress at the physicochemical level, reducing the physiological demand for massive SOD production. In particular, in roots, SOD activity significantly decreased by 38.6% at 5% DTM and by 43.9% at 10% DTM compared with the Control+ group. The maximum reduction in root biomass was observed at 20% DTM, which was 45.6% lower than that of the control. A similar pattern was observed in the shoots, although the activity levels remained consistently higher than those in the roots. SOD activity declined by 17.7% at 5% DTM and reached its maximum suppression of 32.3% at 10% DTM. At 20% DTM concentration, a slight increase was noted compared to the 10% treatment, resulting in a 27.4% reduction relative to the control. These results confirm that 10% and 20% DTM are the most effective in stabilizing shoot metabolism under freezing stress.

#### 3.5.2. Catalase (CAT) Activity

In the Control+ group, CAT activity in the shoots was markedly suppressed, indicating profound damage to the enzymatic apparatus by excess reactive oxygen species (ROS) during hypothermia. The application of DTM demonstrated a “metabolic resuscitation” effect; while the overall physiological demand for antioxidant enzymes decreased, DTM specifically helped to maintain or restore CAT functionality. In roots, CAT activity showed a clear downward trend as DTM concentration increased, dropping from 0.029 to 0.012 μmol H_2_O_2_ · min^−1^ · mg^−1^ protein. This represents a significant reduction of 58.6% at the highest concentration, reflecting a sharp decline in peroxide-induced stress. In shoots, CAT activity remained consistently lower than that in roots but exhibited a different dynamic. Compared to the suppressed levels in the Control+ group, the use of 20% DTM allowed for a narrower but more stable range of activity (0.007–0.018 μmol H_2_O_2_ · min^−1^ · mg^−1^ protein). Notably, across all treatments, root tissues consistently exhibited higher catalase activity than shoots, suggesting that the primary site of peroxide neutralization in *Hordeum vulgare* L. under these conditions is the root system.

#### 3.5.3. Ascorbate Peroxidase (APX) Activity

APX activity exhibited distinct organ-specific responses to DTM treatment (Figure 6C). In roots, APX activity remained remarkably stable, showing only a marginal decrease of 10.6% at the highest DTM concentration, suggesting a low sensitivity of the root ascorbate-glutathione cycle to this specific treatment. In contrast, the shoot tissues displayed a pronounced sensitivity to DTM. Compared to the peak activity in the Control+ group, the introduction of 5% DTM led to a dramatic 88.9% reduction in APX activity. Interestingly, as the DTM concentration increased to 10% and 20%, a partial dose-dependent recovery was observed, with activity levels rising to 0.05 and 0.08 μmol ascorbate (min^−1^ · g^−1^), respectively. Despite this recovery, these values remained 70.5–81.5% lower than those of the control. This trend suggests that while DTM significantly alleviates the initial oxidative pressure in shoots, higher concentrations may prime the enzymatic antioxidant system for a more active but controlled stress response.

#### 3.5.4. Total Protein Content

Dynamic protein content is an important indicator of the plant’s adaptive strategy under stress conditions (Figure 6D). In the Control+ group, barley showed a clear stress response, where the soluble protein level in the shoots increased to 8.01 mg/g FW, which was a sharp increase compared to the negative control (Control−, 1.2 mg/g FW). This accumulation is logically due to the rapid synthesis of cold shock proteins and molecular chaperones.

The application of DTM shifted the plant to a balanced metabolic state. The protein content in the shoots decreased by 16.2% at 5% DTM, by 27.3% at 10% DTM, and by 53.1% at 20% DTM. This downward trend suggests that DTM treatment may alleviate severe environmental stress, allowing the plant to reduce intensive energy expenditure. The strong correlation between the reduction in antioxidant enzyme activities (SOD and APX) and the stabilization of protein levels supports the hypothesis of a potential energy-saving adaptive strategy in the plant. However, further targeted molecular and proteomic analyses are needed to definitively confirm this metabolic optimization.

#### 3.5.5. Generation of ROS

**Figure 7:**
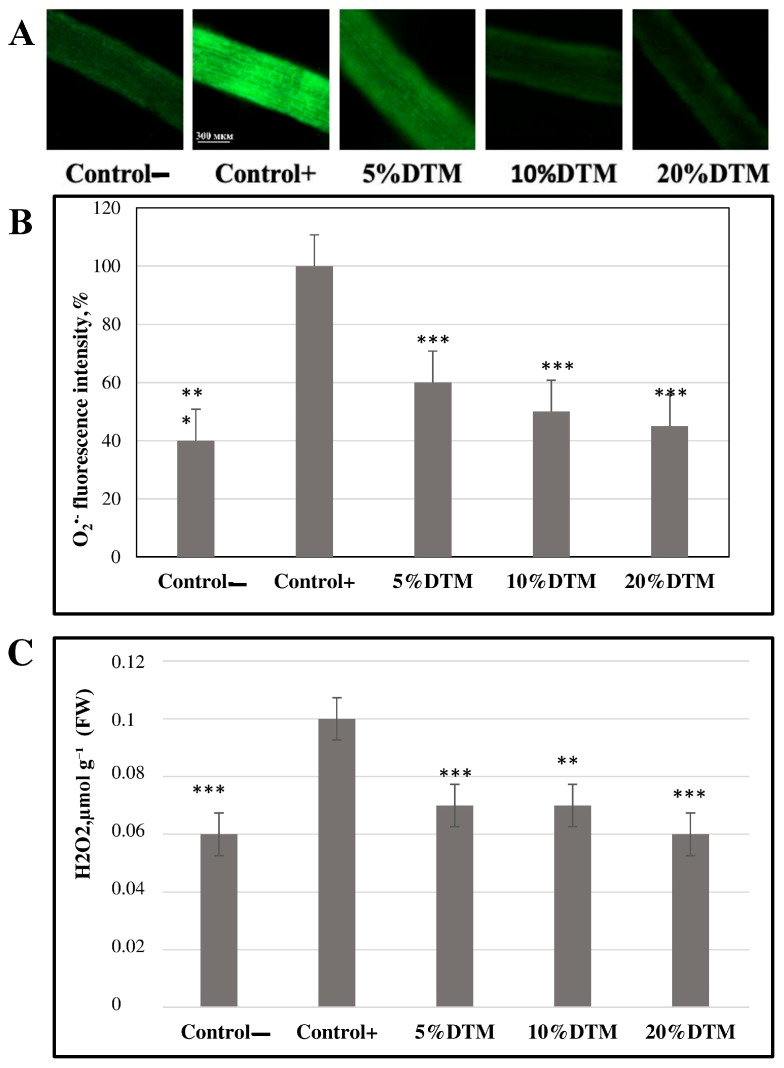
Superoxide and hydrogen peroxide generation in *Hordeum vulgare* L. roots. (**A**) Representative micrographs of roots stained with dihydroethidium (DHE); (**B**) Average DHE fluorescence intensity as a measure of O_2_^·−^ generation; (**C**) Generation of H_2_O_2_. Data are presented as mean ± SE (*n* = 20). Asterisks indicate statistically significant differences from the positive control (Control+) according to a one-way ANOVA (* *p* ≤ 0.05, ** *p* < 0.01, *** *p* < 0.001).

The analysis was conducted by assessing the intensity of specific fluorescence in the root tissue. The roots of the Control+ group were characterized by intense fluorescence over the entire area, indicating massive radical generation (Figure 7A). In the DTM groups, fluorescence intensity gradually decreased. Stress increased fluorescence intensity by up to 100%. The use of DTM at concentrations of 5%, 10%, and 20% reduced this indicator to 60%, 50%, and 45%, respectively (Figure 7B). A sharp increase in hydrogen peroxide levels was observed (from approximately 0.06 to 0.10 μmol g ^−1^) (Figure 7C).

This confirms that stress causes significant production of reactive oxygen species (ROS), which can damage cell membranes. Even 5% DTM significantly reduced hydrogen peroxide levels compared to the positive control (0.10 to 0.07). When the DTM concentration was increased to 20%, the level dropped to 0.06, which was almost identical to that of the negative control (normal plant conditions). The graph shows a clear trend: the higher the DTM concentration, the more effectively it neutralized the oxidative stress. DTM acts as an effective antioxidant, protecting plants from oxidative damage. Concentrations of 10% and 20% DTM were optimal for maintaining oxidative homeostasis near physiological norms.

### 3.6. Mechanism of Diatomite Integration

**Figure 8:**
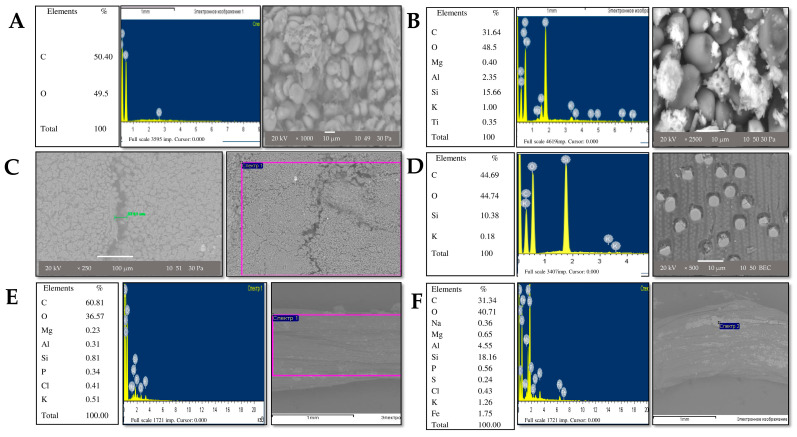
SEM–EDS analysis of diatomite integration into barley seeds after priming treatment: (**A**) control (untreated seeds); (**B**–**D**) seeds treated with 10% DTM; (**C**,**D**) microcracks and natural channels on the seed surface (diameter ~33.5 μm, length ~132 μm); (**E**) control (untreated root); (**F**) Si accumulation in root tissues. Images were obtained at magnifications of up to ×2000 (*n* = 10).

Scanning electron microscopy (SEM–EDS) analysis revealed the physical integration of diatomite particles into seed structures following priming, supporting their role as a protective barrier against cold stress. The experiment was repeated ten times (Figure 8).

Scanning Electron Microscopy (SEM) and Energy-Dispersive X-ray Spectroscopy (EDS) at low magnification (Figure 8C,D) clearly demonstrated the presence of microcracks and natural channels in the seed coat and endosperm, which appear to serve as pathways for diatomite particle penetration into internal seed tissues. The process begins with water absorption (priming), which causes a sharp increase in the hydrostatic pressure within the seed. This leads to the controlled expansion of natural channels and the formation of a network of microcracks in the seed coat and the endosperm. As a result of tissue hydration, the pore diameters reached the critical values of 10–35 µm. These dimensions ideally match the geometry of diatomite particles (1–5 µm), transforming cracks into “transport arteries” for the penetration of silicon structures into the seed. At high magnification (Figure 8B), it was confirmed that the particles were not simply retained on the surface but migrated into the deeper layers of the endosperm. Moreover, the highly ordered nanoporous structure of the diatom frustules was completely preserved, demonstrating their high mechanical resistance to stress during the priming process. This distribution suggests the formation of a structural matrix in the seeds. Comparative observations between untreated and treated seeds (Figure 8A,B) revealed a close association between diatomite particles and starch granules in the treated seeds. The particles appeared to adhere to the storage structures, indicating a potential interaction with reserve compounds.

SEM–EDS analysis demonstrated substantial silicon accumulation in seeds treated with diatomite compared with the control. Silicon content increased from 0% to 15.66% (Figure 8A,B) after priming treatment with 10% DTM. Silicon signals were detected within the internal seed tissues, suggesting the penetration of diatomite-derived silica into the endosperm during the priming process. These findings indicate that seed priming with diatomite may facilitate Si uptake and internal distribution, potentially contributing to enhanced antioxidant protection, reduced oxidative damage, and improved seedling growth in barley plants. In addition to silicon, the seeds contain several other beneficial elements that support plant growth and development and help ensure a strong initial growth stage. Elements such as Mg—0.4%, K—1%, and Ti—0.35% are essential for important physiological and metabolic functions in plants. In addition to integration at the seed stage, the developing root system exhibits a pronounced capacity for DTM. Micrographs (Figure 8E,F) show that the primary roots actively absorbed fine particles through the epiblema and pores in the root cap. According to the results, this indicator increased from 0.8% Si to 18.6% Si.

### 3.7. Interaction of Diatomite Surface with the ROS

**Figure 9:**
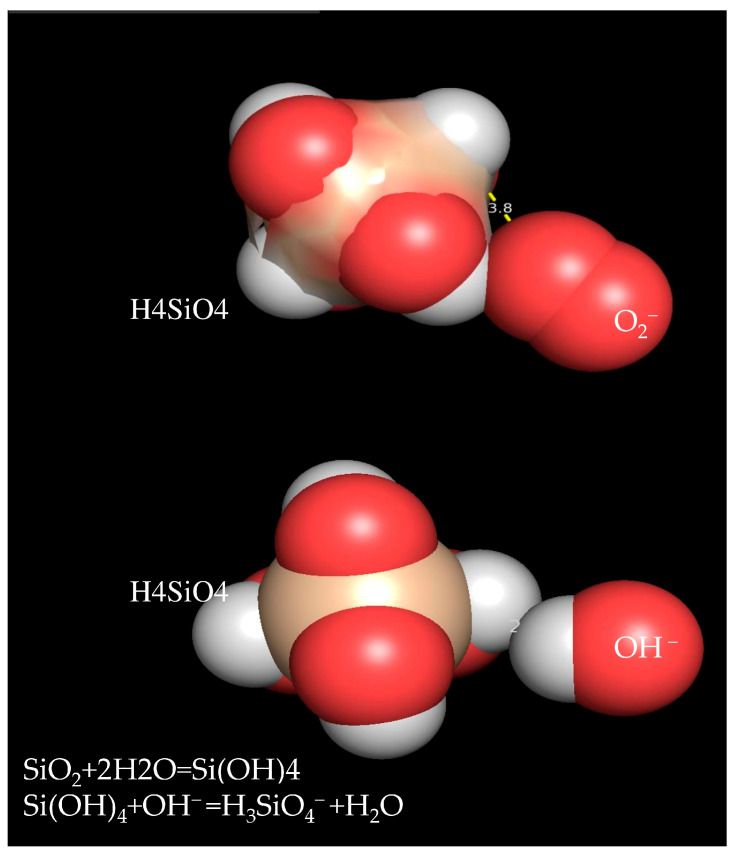
Interaction of diatomite surface with the ROS.

For the H_4_SiO_4_+O_2_^·−^ system, a distance of 3.8 Å was recorded (Figure 9), which corresponds to the initial stage of electrostatic interaction and radical stabilization. For the H_4_SiO_4_^+^OH^−^ system, the distance decreases to 2.2 Å, which is characteristic of a chemical substitution/deprotonation reaction with the formation of the H_3_SiO_4_^−^ anion and a water molecule.

The ability of diatomite and its derivatives to effectively bind ·OH (the most dangerous radical during cold stress) makes it a powerful antioxidant that prevents the irreversible destruction of plant proteins and DNA. The image confirms the mechanism by which silicic acid acts as an active reagent, neutralizing hydroxide ions and potentially affecting the activity of oxygen-free radicals. Based on the provided molecular modeling data and biochemical properties of silicon, the following key aspects of plant protection can be identified: chemical transformation: upon entering the soil, DTM (SiO_2_) hydrates to orthosilicic acid Si(OH)_4_, which is a bioavailable form for root uptake by plants. The interaction of silicic acid with hydroxide ions leads to the formation of silicate ions (H_3_SiO_4_^−^), which are actively involved in cellular metabolism and signaling. As shown in the image, silicon molecules can bind to superoxide radicals (O_2_^·−^), as evidenced by negative Gibbs free energy values (ΔG < −2.0), indicating an efficient catalytic process for ROS scavenging [40]. It is critical to note that since empirical force-field methods (such as UFF in Avogadro) lack quantum mechanical rigor, this value should not be interpreted as an absolute thermodynamic binding energy. This study requires further and additional research.

## 4. Discussion

Our study demonstrates that DTM has protective effects on *Hordeum vulgare* L. under freezing stress, influencing both physiological performance and ultrastructural organization of the plant. Stimulation of root growth and biomass accumulation at 10% and 20% DTM shows that Si-based amendments enhance early plant development [41].

Notably, 10% DTM resulted in optimal growth stimulation, whereas 20% DTM resulted in a slight decline in growth parameters. This pattern is consistent with the concept of an “optimal range” for mineral amendments, where moderate concentrations improve rhizosphere conditions, whereas excessive amounts may alter substrate porosity and the water–air balance. Nevertheless, even at 20% DTM, all growth parameters remained higher than those of the control, confirming the absence of phytotoxic effects and indicating the physiological safety of this material.

SEM–EDS analysis revealed improved preservation of the leaf mesophyll structure under 5–20% DTM treatment, with reduced intercellular spaces and better cellular integrity than that in the control. Concentrations of 10% and 20% DTM optimally supported cellular stability under freezing conditions.

Freezing stress at −3 °C induced a marked reduction in Chlorophyll a, Chlorophyll b, and carotenoid levels in control plants. Freezing-induced membrane destabilization and inhibition of Chlorophyll biosynthetic enzymes typically lead to pigment degradation, largely mediated by excessive accumulation of reactive oxygen species (ROS) and lipid peroxidation in thylakoid membranes [40]. Carotenoids, which play a crucial photoprotective role in dissipating excess energy and preventing photooxidative damage, were also significantly reduced, indicating impairment of the antioxidant defense system [42]. In contrast, DTM-treated plants maintained significantly higher levels of chlorophyll a and carotenoids, suggesting reduced photoinhibition and improved stability of photosynthetic machinery. These findings are consistent with those of previous studies reporting that Si enhances chloroplast membrane stability, reduces ROS production, and maintains photosystem I and II activity under stress conditions [43]. The relatively weaker response of chlorophyll b may be attributed to its structural role in light-harvesting complexes and its comparatively higher stability, as previously reported in cold-tolerant cereal species.

A marked reduction in proline accumulation under DTM treatment, including undetectable levels at 10–20%, indicates a substantial alleviation of stress conditions. Proline is widely recognized as a universal marker of osmotic and oxidative stress, and its accumulation typically reflects cellular dehydration and overproduction of ROS. However, in the present study, reduced proline levels were accompanied by improved growth performance and higher pigment stability, indicating recovery rather than metabolic suppression.

This observation is consistent with reports in which silicon or antioxidant treatments reduced proline accumulation as a consequence of restored redox homeostasis, rather than impaired metabolism [44]. The decreased requirement for proline synthesis likely reflects reduced ROS production and stabilization of cellular water balance. Additionally, enhanced proline catabolism in the mitochondria may contribute to energy recycling during recovery from stressful conditions [45]. Importantly, the simultaneous improvement in physiological performance confirmed that reduced proline accumulation reflects a transition from stress-response metabolism to a restored physiological state rather than metabolic inhibition.

The coordinated changes in total protein content and antioxidant enzyme activities (SOD, CAT, and APX) indicate that DTM does not exert toxic effects but rather induces adaptive metabolic reprogramming in cells.

A significant reduction in the total protein content in both roots and shoots reflects a decreased demand for stress-induced proteins, including heat shock proteins and antioxidant enzymes, which are typically upregulated under oxidative stress [46]. The parallel decrease in protein content and enzyme activity suggests reduced ROS accumulation and a lower requirement for stress defense mechanisms, resulting in improved metabolic efficiency in the treatment group.

Catalase activity showed a dose-dependent decrease, particularly in roots, where it plays a key role in detoxifying the hydrogen peroxide generated by intense respiratory activity [47]. The gradual reduction in CAT activity indicates a decreased intracellular H_2_O_2_ load rather than enzyme inhibition [48,49].

SOD is a key enzyme in the first line of antioxidant defense, dismutating the highly toxic superoxide radical (O_2_) into hydrogen peroxide (H_2_O_2_). Silicon (Si) from DTM is deposited in the cell walls and under the cuticle, forming a silicon–cellulose double layer. This increases the mechanical rigidity of tissues and reduces the likelihood of ice crystals damaging membranes [50]. DTM is believed to neutralize reactive oxygen species, reducing oxidative stress and the need for antioxidant protection, which contributes to energy conservation and improved plant growth and development under cold stress conditions.

APX activity exhibited organ-specific responses: while roots maintained relatively stable activity, shoots showed a pronounced decrease, even at low DTM concentrations. This indicates a reduced requirement for high-affinity H_2_ scavenging systems in photosynthetic tissues, which is consistent with an improved redox balance. Together, these results suggest a functional reconfiguration of the antioxidant system, in which energy-intensive pathways are replaced with more stable and efficient regulatory mechanisms.

The observed biochemical and physiological responses were strongly supported by the SEM-based structural evidence. Three complementary protective mechanisms have been proposed [51,52,53,54].

First, the nanoporous structure of the diatom frustules creates a thermal insulation effect. Air trapped within siliceous pores reduces thermal conductivity (typically 0.0984 W/m · K), thereby slowing intracellular cooling rates and limiting the formation of ice crystals during freezing stress. This stagnant air layer acts as a thermal barrier that disrupts the rapid transfer of cold air, thereby creating a stable microenvironment within the seed tissues [18,19,55]. Based on the structure of the DTM, we hypothesize that nanopore- mediated insulation may contribute to thermal protection in barley plants during freezing stress. However, as direct thermophysical measurements were not performed in this study, this mechanism remains a plausible theoretical model that requires further experimental validation.

Second, diatomite particles fill intercellular spaces and microcracks, forming a rigid structural scaffold that stabilizes the cellular architecture under dehydration and freezing-induced contraction.

Third, the high adsorption capacity of diatomite contributes to the formation of a hydrophilic–adsorptive barrier that regulates water distribution and prevents osmotic shock during freezing-induced dehydration [56].

Collectively, these results indicate that diatomite functions as a multifunctional protective agent by combining physical, structural, and biochemical effects on the skin. At the physical level, it modifies seed and soil microenvironments; at the structural level, it reinforces tissue integrity; and at the biochemical level, it modulates redox balance and metabolic activity. The convergence of these mechanisms results in improved growth performance and enhanced cold stress tolerance in *Hordeum vulgare* L.

## 5. Conclusions

Diatomite (DTM) application enhanced barley growth, particularly root development and biomass accumulation, with the most effective response observed at 10% and 20% concentrations of DTM (concentrations higher than 20% do not have a toxic effect but may cause unexpected effects, highlighting the limitation of a complete dose–response curve beyond general observations of the substrate’s properties). A concentration of 10% DTM is optimal for vegetative growth under non-stressed or moderately stressed conditions. In contrast, 20% DTM induces a strong defense/adaptation response, which enhances physical defense and increases frost tolerance, although a slight decrease in maximum growth potential was observed. Crucially, DTM treatment significantly reduced the accumulation of reactive oxygen species (ROS) during the initial stages of stress. By directly alleviating oxidative damage through its structural and protective properties, diatomaceous earth effectively shields the cellular environment of the cells. Consequently, this leads to the downregulation of the antioxidant enzyme system and a decrease in proline accumulation. By reducing the ROS burden and optimizing the plant energy budget, DTM serves as an effective and safe soil amendment that improves plant performance under cold-stress conditions.

## Data Availability

The original contributions presented in this study are included in the article. Further inquiries can be directed to the corresponding author.

## Abbreviations

The following abbreviations are used in this manuscript:

DTM	Diatomite
SOD	Superoxide dismutase
CAT	Catalase
APX	Ascorbate peroxidase
ROS	Reactive oxygen species
Chl	Chlorophyll
